# Application of Biotechnological Techniques Aimed to Obtain Bioactive Compounds from Food Industry By-Products

**DOI:** 10.3390/biom11010088

**Published:** 2021-01-12

**Authors:** Jose Antonio Curiel

**Affiliations:** Research and Development of Functional Food Centre (CIDAF), PTS Granada, Avda. Del Conocimiento 37, Bioregión Building, 18016 Granada, Spain; jacuriel@cidaf.es

Currently, food losses represent a serious imbalance in the dimensions of availability and accessibility in the global food system in the short term. Therefore, food waste management plays a central role in this system, as food waste is expected to increase to around 126 Mt by 2020 [1], and the slogan “from waste to resources” is being promoted to order to inject food waste into the economy as new raw materials, thus increasing security of supply. Since up to 42% of food waste occurs in household activities and 39% in the food industry [1], the circular economy focuses on the revaluation of those food losses (low-cost raw materials). These “secondary raw materials” can be traded and transported as primary raw materials of traditional extractive resources, thus accentuating certain properties (nutritional, functional, antimicrobial, etc.) through different recycling systems which make treatment of this by-product economically attractive for the food industry [2].

Because of the different food by-products (shells, bones, seeds, viscera, etc.) being discarded by the food industry, the scope of this Special Issue is focused on those new challenges currently being developed through the application of specific methodologies for each of them in order to achieve their targeted valorization based on the compounds to be highlighted.

In this sense, and regarding seed valorization, Merah et al., 2020 [3], described a novel biorefinery methodology applied to cumin (*Cuminum cyminum*) seeds from different geographical origins in order to evaluate oil yield as well as the valorization of remaining by-products by investigating their nutritional content and biological activity. These authors achieved vegetable and essential oils enriched in proteins and sugar, regardless of their origin, exhibiting high levels of petroselinic fatty acid and sterols. On the other hand, cakes and aromatic water also presented high levels of proteins, fibers, sugars, and phenols, revealing interesting antioxidant and antibacterial activities. Therefore, Merah et al., 2020’s results contributed and emphasized the valorization of cumin and its by-products toward health promoting compounds under a biorefinery concept.

On the other hand, high amounts of food by-products concern fruit pits. In this regard, Kawabata et al., 2020 [4], attempted to solubilize the wood-like carapace (stone) encasing of the pit by subcritical fluid extraction with the aim of extracting useful chemicals. The degrees of solubility for various treatments (190 °C; 3 h) were determined as follows: subcritical water (54.9%), subcritical 50% methanol (65.5%), subcritical 90% methanol (37.6%), subcritical methanol (23.6%), and subcritical isopropyl alcohol (14.4%). Syringaldehyde, sinapyl alcohol, coniferyl alcohol methyl ether, sinapyl alcohol methyl ether, 5-(hydroxymethyl)-2-furfural, and furfural were present in the subcritical 90% methanol extract. Their findings provided a solubilization method that allows the main phenolic constituents of the pits to be extracted under mild conditions.

In addition to seeds or fruit pits, great interest exists in hard shells because of their high volume as a vegetable by-product. Concerning that, Villasante et al., 2020 [5], explored the effects of the extrusion temperature and screw speed on the moisture content, water and oil absorption index, water solubility index, color, phenolic compounds, condensed tannin compounds, and antioxidant activity of pecan nut shell extrudates. Extrusion, performed at 70 °C and 150 rpm, almost doubled the concentration of polyphenols in the non-extruded shell and significantly increased radical scavenging activity. Extrusion significantly increased most phenolic acid compounds, including gallic acid, ellagic acid pentose, ellagic acid, dimethyl ellagic acid rhamnoside, and dimethyl ellagic acid. The soluble fiber in extrudates was more than three-fold higher than in the control. Therefore, extrusion at 70 °C and 150 rpm increased the concentration of phenolic compounds, anti-oxidant activity, and total dietary and soluble fiber. Their findings support the notion that extruded pecan nut shells can be used in clean-label products and improve their nutraceutical value.

Other interesting by-products correspond with the industrial fatty side streams, resulting from obtaining oil. In this context, Papadaki et al., 2020 [6], described the oleogelation of extra virgin olive oil using different wax ester concentrations derived from soybean fatty acid distillate, a by-product of industrial soybean oil refining. Analyses of the mechanical properties of oleogel showed a firmness of 3.8 N, which was then reduced to around 2.1–2.5 N during a storage period of 30 days at 4 °C. Therefore, their results showed that wax esters derived from soybean fatty acid distillate was successfully utilized for the oleogelation of olive oil, resulting in a novel oleogel with desirable properties for food applications.

Finally, regarding vegetal by-products, it is highlight that certain crops are increasing yearly and consequently the volume of by-products as well, such as wheat bran. In this sense, Katileviciute et al., 2020 [7], have collected recent information about different methods and target compounds valorized from this abundant by-product.

Beyond vegetable by-products, the approaches aimed at valorizing animal food matrices such as meat and fish residues are also a current trend. Concerning that, Premetis and Labrou, 2020 [8], reported the development of a cellulose-based affinity adsorbent isolated from recycled newspapers and triazine dye Cibacron Blue 3GA as ligand able to purify proteases from fish by-products. The affinity adsorbent was applied for the development of a purification procedure for proteases from gilt-head bream (*Sparus aurata*) by-products such as stomach and pancreas. A single-step purification protocol for trypsin and chymotrypsin was developed and optimized. These authors developed an interesting protocol capable of obtaining proteases with high yields suitable for technical and food industry purposes. Continuing with fish by-products, Vázquez et al., 2020 [9], focused their studies on the valorization of wastes generated in the processing of farmed fish. In their study, turbot (*Scophthalmus maximus*) by-products were subjected to Alcalase hydrolysis. All the fish protein hydrolysates showed a high yield of digestion (>83%), very remarkable degrees of hydrolysis (30–37%), high content of soluble protein (>62 g/L), an excellent profile of amino acids, and almost total in vitro digestibility (higher than 92%). These authors concluded that viscera hydrolysates exhibited the most antioxidant and antihypertensive activities.

In the same vein (non-vegetal by-products), valorization of dairy products keeps improving through the obtaining of attractive ingredients. In this sense, Musatti et al., 2020 [10], have recently described the application of cheese whey permeate-based culture medium for *Lactobacillus sakei* with the aim of producing food-grade sakacin A, a bacteriocin exhibiting a specific antilisterial activity. The most convenient formulation was liquid cheese whey permeate supplemented with meat extract (4 g/L) and yeast extract (8 g/L). Their results demonstrate the feasibility of producing sakacin A from cheese whey permeate with a significant reduction of 70% compared to the corresponding costs with Man, Rogosa and Sharpe (MRS) medium. Taking into account that the limited use of bacteriocins for food application is mainly due to the high production cost, these authors contribute to widening the range of applications of sakacin A as antilisterial agent.
